# The effect of prophylactic mesh implantation on the development of incisional hernias in patients with elevated BMI: a systematic review and meta-analysis

**DOI:** 10.1007/s10029-022-02675-x

**Published:** 2022-09-14

**Authors:** F. Pianka, A. Werba, R. Klotz, F. Schuh, E. Kalkum, P. Probst, A. Ramouz, E. Khajeh, M. W. Büchler, J. C. Harnoss

**Affiliations:** 1 Department of General-, Visceral- and Transplantation Surgery, University of Heidelberg, Im Neuenheimer Feld 420, 69120 Heidelberg, Germany; 2 Study Center of the German Surgical Society, University of Heidelberg, Heidelberg, Germany; 3 Department of Surgery, Cantonal Hospital Thurgau, Frauenfeld, Switzerland

**Keywords:** Incisional hernia, Obesity, BMI, Prophylactic mesh, Systematic review

## Abstract

**Background:**

Incisional hernia is a common complication after midline laparotomy. In certain risk profiles incidences can reach up to 70%. Large RCTs showed a positive effect of prophylactic mesh reinforcement (PMR) in high-risk populations.

**Objectives:**

The aim was to evaluate the effect of prophylactic mesh reinforcement on incisional hernia reduction in obese patients after midline laparotomies.

**Methods:**

Following the PRISMA guidelines, a systematic literature search in Medline, Web of Science and CENTRAL was conducted. RCTs investigating PMR in patients with a BMI ≥ 27 reporting incisional hernia as primary outcome were included. Study quality was assessed using the Cochrane risk-of-bias tool and certainty of evidence was rated according to the GRADE Working Group grading of evidence. A random-effects model was used for the meta-analysis. Secondary outcomes included postoperative complications.

**Results:**

Out of 2298 articles found by a systematic literature search, five RCTs with 1136 patients were included. There was no significant difference in the incidence of incisional hernia when comparing PMR with primary suture (odds ratio (OR) 0.59, 95% CI 0.34–1.01, *p* = 0.06, GRADE: low). Meta-analyses of seroma formation (OR 1.62, 95% CI 0.72–3.65; *p* = 0.24, GRADE: low) and surgical site infections (OR 1.52, 95% CI 0.72–3.22, *p* = 0.28, GRADE: moderate) showed no significant differences as well as subgroup analyses for BMI ≥ 40 and length of stay.

**Conclusions:**

We did not observe a significant reduction of the incidence of incisional hernia with prophylactic mesh reinforcement used in patients with elevated BMI. These results stand in contrast to the current recommendation for hernia prevention in obese patients.

## Introduction

Incisional hernia (IH) is a common complication after midline laparotomy. The incidence can reach up to 40–70% with 104.000 estimated cases per year in Germany alone [1–4]. Despite the additional costs of hernia repair surgery, IH can lead to a significant quality of life reduction including the impending risk of major complications and mortality [5, 6].

Although risk factors, such as chronic obstructive pulmonary disease (COPD), long-term steroid use and infections, are contributing to the formation of IH, obesity and connective tissue disorders are considered the most influential [7, 8].

After midline laparotomy approximately one-third of patients with a BMI ≥ 27 kg/m^2^ develop an IH [9]. Obesity is associated with a higher intraabdominal pressure leading to increased stress on the abdominal wall [10]. Moreover, due to a hypoxic wound environment in adipose tissue and a thereby decreased expression of vasculogenic cytokines wound healing is further impaired [11–13].

Despite different operation methods, suture materials and mesh augmentations currently used for IH repair, the recurrence rates considering a median follow-up of at least 75 months after suture repair remain up to 63% and 32% after mesh repair, respectively [14]. Furthermore, once an IH occurs, subsequent repairs are associated with an increased risk of major complications and health care costs [15]. Therefore, prevention of IH is of likely underestimated economic importance as well as for patients’ safety [16, 17].

Many studies have evaluated mesh reinforcement for IH repair but only few studies have investigated the prophylactic use in midline laparotomy [8, 18–21]. According to the European Hernia Society (EHS) Guidelines, the level of recommendation for the standard use of prophylactic mesh reinforcement (PMR) remains low in risk groups [22]. In a large multicentre randomised controlled trial (RCT) including 480 patients Jairam et al. achieved a significantly lower IH rate of 13% in the prophylactic onlay group versus 30% in the primary suture group (OR 0.37) after midline laparotomy in patients with AAA or increased BMI. Nevertheless, in the subgroup of obese patients alone, no significant effect of PMR could be shown [8].

Several other studies analysed PMR in patients with elevated BMI with conflicting results, but there is still a lack of high-quality evidence regarding effectiveness and safety, and therefore, recommendations are still controversially discussed [1, 8, 18–21].

The aim of this systematic review and meta-analysis was (i) to summarise and critically appraise the available evidence on PMR in obese patients to allow for recommendations in clinical practice and prospective research and (ii) to identify important risk subgroups. The results can, furthermore, serve as a reference for sample size calculation in future trials.

## Methods

This systematic review was conducted according to the recommendation of the Preferred Reporting Items for Systematic reviews and Meta-Analyses (PRISMA) guidelines [23], the recommendations from the Study Center of the German Society of Surgery [24] and as outlined in a predefined protocol (PROSPERO 2021: CRD42021237872). All stages of study selection, data abstraction, and quality assessment were carried out independently by two reviewers (A.W. and F.P.). Any disagreements were resolved by consulting a third reviewer (J.C.H).

### Systematic literature search

MEDLINE (via PubMed), Web of Science and the Cochrane Library were systematically searched for relevant studies. No language restrictions were applied. Reference lists of relevant studies were searched manually and the ‘‘related articles’’ function in Pub Med was used. The search strategy combined text words and MeSH terms related to mesh versus no mesh for incisional hernia prevention:

(Mesh*[tiab] OR "Surgical Mesh"[Mesh]) AND (((incision*[tiab] OR ventral*[tiab]) AND hernia*[tiab]) OR "Incisional Hernia"[Mesh]) AND (random*[tiab] OR RCT*[tiab] OR "Randomized Controlled Trial"[pt] OR "Randomized Controlled Trials as Topic"[Mesh] OR "Controlled Clinical Trial"[pt] OR "Clinical Trials as Topic"[Mesh] OR "controlled Trial"[tiab] OR "clinical Trial"[tiab] OR "controlled study"[tiab] OR group*[tiab] OR "Control Groups"[Mesh] OR "Prospective Studies"[Mesh] OR control[tiab] OR controls[tiab] OR (prospectiv*[tiab] AND (controlled[tiab] OR matched[tiab]))) NOT (animals [mh] NOT humans [mh]). Corresponding search strategies were used for Web of Science and for the Cochrane Library. The detailed search strategy is freely accessible in the protocol (PROSPERO 2021: CRD42021237872).

### Study selection

For sensitivity reasons following the ‘‘best evidence approach’’, only available randomised controlled trials (RCT) were included. Only RCTS reporting BMIs, surgical procedures, and mesh placement methods were included. All studies containing pooled data on regular BMI patients, children, and abdominal aortic aneurysms (AAA) were excluded. Further exclusion criteria were previous midline laparotomy, previous IH, emergency laparotomy, immunosuppressive therapy, and interlay mesh position. Titles and abstracts were screened independently by two reviewers and full text articles were obtained when inclusion criteria were met. If two or more publications reported on the same patient population, the study with the most comprehensive and recent data was used.

### Outcome parameters

The definitions of outcome parameters are summarized in Table 1 (definitions of investigated outcomes). The following outcomes were assessed:

1. Effectiveness (incidence of IH)
2. Safety (postoperative complications, adverse events of PMR)
3. Length of hospital stay

**Table 1:** Definitions of investigated outcomes

Effectiveness	Incidence and success of incisional hernia prevention by prophylactic mesh reinforcement (PMR)
Safety	Postoperative complications (Incidence of surgical site infections, seroma) and adverse events
Length of hospital stay	Number of days of hospital stay

### Data extraction

The digital data extraction sheet comprised the following predefined items: (i) study identification (first author and year of publication); (ii) essential study data (study design, recruitment and follow-up period, treatment arms, number of subjects); (iii) baseline characteristics of study subjects (mean age, sex, BMI, etc.); and (iv) quality features. Finally, the outcome parameters described above were extracted for individual treatment groups as far as reported. Baseline comparability of the different treatment groups was evaluated.

### Risk of bias and quality of evidence

Risk of bias was assessed using the Cochrane risk-of-bias tool RoB2 [25]. The tool calculates the overall risk of bias according to five domains including: randomization process, deviation from intended interventions, missing outcome data, measurement of the outcome and selection of the reported result. Risk of bias judgement was defined in three grades: low, some concerns and high [25]. A rating of the certainty of evidence for every outcome was made using the GRADE approach [26], investigating for inconsistency, indirectness, and imprecision.

### Statistical analysis

Meta-analyses were calculated using R version 4.0.3. Intervention effects were estimated via relevant outcome parameters from the included trials. An estimation of odds ratios (OR) and associated 95% confidence intervals for dichotomous data (IH and morbidity) was created with the Mantel–Haenszel model. For continuous data, weighted mean differences (MD) and associated 95% confidence intervals were calculated using an inverse-variance model. Means and standard deviations, if not reported, were estimated [27]. A two-sided level of significance with *p* < 0.05 was considered statistically significant. Trials were evaluated for statistical heterogeneity with the *I*^2^ statistic. Due to clinical heterogeneity among surgical trials, the random-effects model was used as a default [24].

Subgroup analyses were conducted for superficial and deep surgical site infections (SSI), length of stay (LoS) and IH incidence in patients with obesity grade III according to the WHO definition (BMI ≥ 40) [28].

## Results

**Fig. 1:**
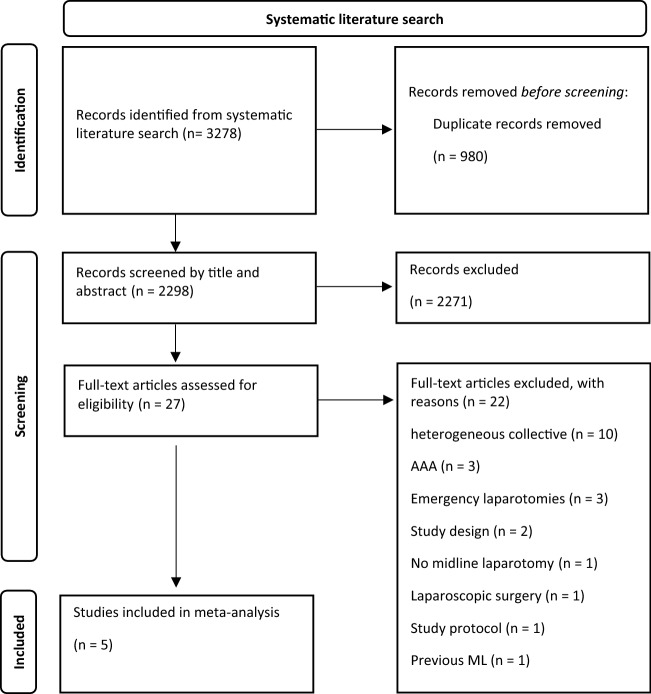
PRISMA flowchart. Abdominal aortic aneurysm (AAA), Midline laparotomy (ML)

A total of 2298 references were identified in the literature search (Fig. 1: PRISMA flowchart). After removing of duplicates and critical evaluation of abstracts, 27 full-text publications were finally assessed for eligibility. After careful review of the full texts, 22 studies did not meet the inclusion criteria due to the following reasons (Fig. 1): heterogenous study collective with normal weight patients (*n* = 10), aortic aneurysms (*n* = 3), emergency laparotomy (*n* = 3), performed by a single surgeon (*n* = 1), study design without proper randomization (*n* = 1), large number of previous laparotomies (*n* = 1), only study protocol (*n* = 1), laparoscopic surgery (*n* = 1), not midline laparotomy (*n* = 1). Authors of potentially suitable trials containing heterogeneous collectives were contacted for individual patient data which were acquired in one trial [8]. Therefore, five RCTs were included for qualitative and quantitative synthesis [8, 18–21].

### Study characteristics

All five trials included obese patients undergoing midline laparotomy. In total, 1.136 patients were allocated to PMR (*n* = 657) or PS (*n* = 479). The patients’ mean age was 45.8 years. In four out of five studies[8, 19–21] the assessment of IH was done by clinical examination combined with ultrasound or CT scan. One study solely used clinical examination [18]. The follow-up in all studies ranged from 24 to 48 months. Two RCTs [20, 21] used sublay PMR exclusively, the three others used either sublay or onlay PMR [8], preperitoneal mesh [19], or intraperitoneal onlay [18]. Concerning mesh material, three studies used a non-absorbable polypropylene mesh [8, 19, 21], one study an absorbable polyglactin mesh [18] and one trial an absorbable biological mesh (Surgisis Gold) [20]. An overview of baseline study characteristics can be found in Table 2.

**Table 2:** Baseline study characteristics

Study	Study type	n (PS/PMR)	FU (months)	FU-method	BMI (mean/SD)/(PS/PMR)	Suture technique	Mesh technique/type	Indication
Abo-Ryia 2013	RCT	32/32	48	PE, US	51.4 (10.5)/52.2 (9.1)	Continuous, non-absorbable	Preperitoneal/Polypropylene	Bariatric surgery
Jairam 2017	RCT	70/260	24	PE, US, CT	31.5 (3.5)/32.4 (5.1)	Continuous, slowly absorbable	Onlay or sublay/Lightweight polypropylene	General surgery
Pans 1998	RCT	144/144	30	PE	43.7 (0.6)/43.8 (0.5)	Interrupted, absorbable	Intraperitoneal/Polyglactin	Bariatric surgery
Sarr 2014	RCT	195/185	24	PE and/or imaging	48.2 (7.7)/48.2 (8.2)	Continuous, non-absorbable, slowly absorbable	Sublay/Bioprosthesis	Bariatric surgery
Strzelczyk 2006	RCT	38/36	28	PE, US	46.8 (7.6)/46.2 (7.1)	Continuous, non-absorbable	Sublay/Polypropylene	Bariatric surgery

*PS* primary suture, *PMR* primary mesh reinforcement, *RCT* randomised controlled trial, *PE* physical examination, *US* ultrasonography, *CT* computed tomography, *SD* standard deviation, *FU* follow-up

### Critical appraisal and risk of bias

Three of five included RCTs reported random sequence generation and an adequate description of allocation concealment, resulting in a low risk of selection bias [8, 20, 21]. Abo-Ryia et al. [19] and Pans et al. [18] randomly assigned patients to either intervention group without clarifying the randomization process or method. Blinding methods were only reported in the studies by Jairam et al. [8] and Strzelczyk et al. [21]. Although blinding of physicians may be difficult or impossible, blinding of patients or outcome assessors would have been feasible, and therefore, detection and performance bias remains unclear in three studies [18–20]. None of the RCTs was considered to have a high risk of attrition bias based on the presented data but only two studies specifically reported losses to follow-up [8, 21]. Outcome data were well-reported in all RCTs, therefore, resulting in a low risk of bias. Definition of endpoints and/or outcome parameters were stated in all studies [8, 18–21]. The applied surgical technique was reported in a standardised manner in all RCTs [8, 18–21]. Two studies [8, 20] registered the study protocol, but only Jairam et al. published said protocol [29]. All the included studies defined and followed the stated primary and secondary endpoints. A summary according to the Cochrane risk-of-bias tool (RoB2) [25] can be found in Tab. 3.

**Table 3:** Risk of bias summary of included studies

	Randomisation process	Deviations from intended interventions	Missing outcome data	Measurement of outcome	Selection of the reported result	Overall
Abo-Riya 2013	!	!	+	−	+	−
Jairam 2017	+	+	+	+	+	+
Pans 1998	!	!	+	−	+	−
Sarr 2014	+	+	+	−	+	−
Strzelczyk 2006	+	+	+	+	+	+

Low risk (+), some concern (!), high risk (−)

## Quantitative analysis

### Prophylactic treatment effectiveness

**Fig. 2:**
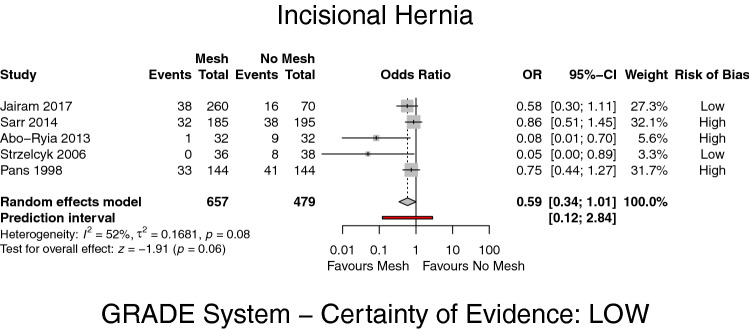
Forest plot of incisional hernia after prophylactic mesh reinforcement versus primary suture

All the included studies reported on the occurrence of IH with a minimum follow-up of at least 24 months: overall 657 patients in the PMR and 479 in the PS group were analysed, with an IH incidence of 14% versus 23% in either group, respectively. There was no significant difference comparing the intervention groups (OR 0.59, 95% CI 0.34–1.01, *p* = 0.06, *I*^2^ = 52%) (GRADE: low) (Fig. 2).

**Fig. 3:**
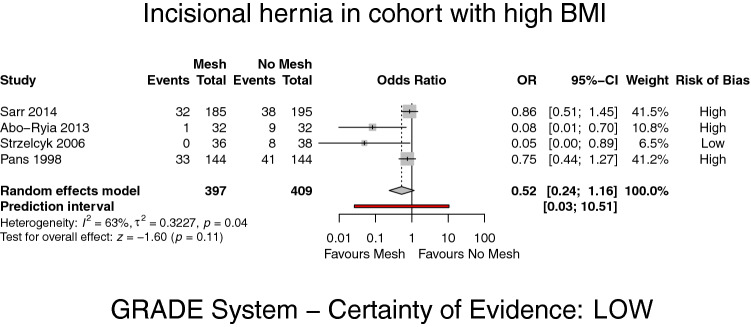
Forest plot of incisional hernia in patients with a BMI ≥ 40 after prophylactic mesh reinforcement versus primary suture

In the subgroup analysis of IH in patients with BMI ≥ 40, four trials [18–21] with a total of 397 patients in the PMR group and 409 patients in the PS group were analysed. There was no significant difference in the incidence of IH (OR 0.52; 95% CI 0.24–1.16, *p* = 0.11, *I*^2^ = 63%) (GRADE: low) (Fig. 3).

### Postoperative complications

**Fig. 4:**
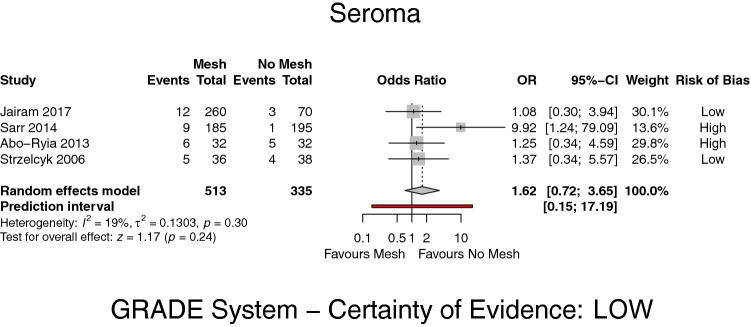
Forest plot of seroma formation after prophylactic mesh reinforcement versus primary suture

Four studies [8, 19–21] reported on the formation of seroma summarizing 513 patients in the mesh group and 335 patients in the PS group. Meta-analysis suggests no significant influence of mesh implantation on the formation of seroma in the investigated collective (OR 1.62, 95% CI 0.72–3.65, *p* = 0.24, *I*^2^ = 19%) (GRADE: low) (Fig. 4).

**Fig. 5:**
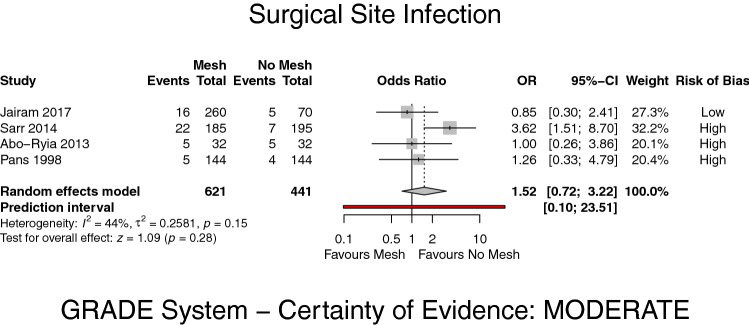
Forest plot of surgical site infections after prophylactic mesh reinforcement versus primary suture

A total of four RCTs [8, 18–20] investigated SSIs. Only one study [8] classified these infections according to the Center of Disease Control and Prevention (CDC) [30]. The remaining trials [18–20] differentiated superficial and deep wound infections from organ space infections in a clear manner. For this analysis superficial and deep wound infections were combined being the most relevant in mesh placements. There was no significant difference in the occurrence of superficial and deep SSI (OR 1.52, 95% CI 0.72–3.22, *p* = 0.28, *I*^2^ = 44%) (GRADE: moderate) (Fig. 5).

### Length of hospital stay

**Fig. 6:**
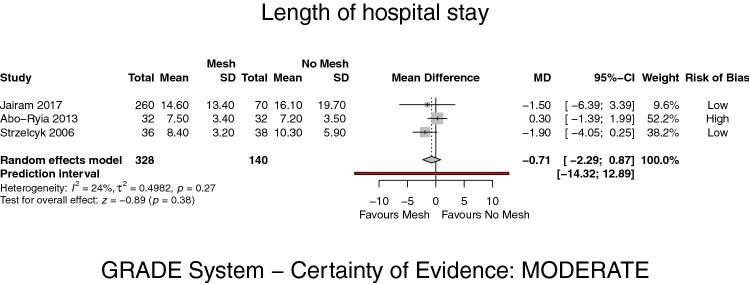
Forest plot of length of hospital stay after prophylactic mesh reinforcement versus primary suture

Three studies [8, 19, 21] reported on LoS. The mean stay for PMR was 10 days and 11 days for PS, respectively. There was no significant difference in the length of hospital stay between groups (MD −0.71, 95% CI −2.29–0.87, *p* = 0.38, *I*^2^ = 24%) (GRADE: moderate) (Fig. 6).

## Discussion

The aim of this study was to investigate the potential benefit of prophylactic mesh placement in patients with an elevated BMI. The mean BMI of all included studies was 44.3, hence this study shows the effect of PMR in patients with mainly obesity grade III [31]. There is no officially recognized BMI limit for mesh implantation recommendation. Jairam et al. initially included patients with a BMI of ≥ 30, but by a trial amendment, the cutoff was reduced to ≥ 27 due to results from the INSECT trial [9], which showed an increased IH incidence in this risk group. Using BMI as a parameter for the necessity of mesh implantation includes inherent deficiencies. BMI measurement only correlates indirectly with body fat and, therefore, shows poor sensitivity and specificity in the quantification of obesity [32]. Nevertheless, there is a lack of feasible, broadly available and easy to use alternatives. Therefore, to ultimately define a BMI limit regarding the risk to develop an IH, further large RCTs and data of national registers are needed.

There was no significant reduction in the incidence of IH when using PMR based on a low certainty level of evidence. Furthermore, subgroup analysis in patients with obesity grade III showed a similar missing effect of PMR with moderate evidence. These results stand in contrast to several RCTs [8, 19, 21, 33–35] and other meta-analyses [1, 15, 36–38]. One of the main factors explaining the different findings is the heterogeneous study population included in RCTs and other meta-analyses, e.g., patients with AAA and emergency operations. Recent evidence shows that individuals undergoing aortic surgery are at an increased risk, up to 37%, for developing IH than patients needing median laparotomy for other reasons [39, 40]. Therefore, investigating a mixed patient collective, leads to an outcome in favour of the prophylactic mesh group and subsequently wrongfully deduced conclusions and recommendations. To minimize selection bias and provide data of high validity, further high-quality multicentric RCTs are needed. This would offer new and undistorted evidence, which is currently limited due to the heterogeneity of the included studies.

Obese patients are already at a higher risk of developing IH. Seiler et al. [9] postulated a hernia rate of 37.2% in patients with BMI ≥ 30. They also frequently suffer from comorbidities, such as pulmonary disorders (chronic obstructive pulmonary disorder, sleep apnea, etc.), diabetes mellitus, reduced mobility, and hypertension further increasing the risk of incisional hernia development [31]. Hence, obesity needs to be taken into account in future trials to identify patients profiting from PMR.

In recent literature five systematic reviews and meta-analyses [1, 15, 36–38] reviewing the effect of PMR were published. The most recent analysis by Depuydt et al. [1] summarized 1633 patients in the mesh reinforcement group and 1533 in the primary suture group. They reported an OR for IH of 0.37 (95% CI, 0.30–0.46, *p* = 0.01, *I*^2^ = 62%). The positive effect for PMR was likely due to a heterogenous study collective and thereby overestimating the effect of PMR in patients with an elevated BMI. The other systematic reviews and meta-analyses [1, 15, 36–38] were also in favour of PMR due to the same reasons. Moreover, these meta-analyses included pooled data of RCTs and non-RCTs, which is not recommended, according to the Cochrane Collaboration [25]. Including non-RCTs increases the risk for distorted results due to selection bias, confounding and large heterogeneity of studies; therefore, the proposed recommendations have to be treated with caution.

This study contains four RCTs investigating bariatric surgery patients [18–21] and one trial comparing a mixed patient collective [8], including patients with a history of cancer, AAA or other diseases undergoing median laparotomy. The latter included a total of 480 patients of which 150 suffered from an AAA and 330 had an BMI ≥ 27. For this analysis, individual patient data were acquired from the authors and only patients with BMI ≥ 27 were included, excluding patients with AAA. This results in a homogenous study collective to adequately investigate the IH risk for obese patients.

Comparing the analysed trials, only one [8] described a suture to wound ratio of 4:1 and small stich technique according to Deerenberg et al. [41]. This closure technique is considered the gold standard for abdominal fascia closure, but the STITCH trial, investigating the matter, was published in 2015 [41]. Since then, no relevant study except the PRIMA trial [8] was published. Although this limits the findings of the previous studies, there is a general lack of RCTs comparing the optimal wound closure to PMR. Currently there is one RCT investigating a combination of small stich technique and onlay mesh reinforcement (OMR) in progress [42].

Especially in patients with OMR there is an increased incidence in subcutaneous fluid formations [8], which is suspected to subsequentially lead to a higher rate of SSI [43]. Although according to Jairam et al. [8] seroma did occur in higher numbers in the OMR group, there were no additional interventions reported for these patients [8]. In this meta-analysis seroma-formation occurred in both cohorts similarly, likely due to a known higher incidence in patients with severe obesity [44], therefore, resulting in no significant difference.

In this meta-analysis, superficial and deep SSIs were summarised using the CDC recommendations [30]. Although only one study [8] specifically declared the use of this classification, all the other included trials clearly differentiated the location, depth and need for intervention concerning SSIs, enabling the use of this easily applicable classification for this analysis. In general, obese patients show a higher incidence of SSIs after median laparotomy [45]. Although the use of a mesh may lead to prolonged wound healing or even reintervention in case of an SSI there is currently no evidence for an increased infection rate using PMR [8].

Four of the included studies [8, 19–21] used an imaging-based follow-up method of at least an ultrasound to detect the formation of an IH. The patients enrolled in the study by Pans et al. [18] were solely examined physically, which is not recommended due to a high rate of non-detected IH, according to the EHS Guidelines [22]. More importantly the follow-up interval in all the analysed trials was longer than 12 months. Fink et al. reported a 12.6% hernia rate after 1 year postoperatively, but 22.4% after 3 years [46]. This shows that the presented meta-analysis contains viable data concerning the occurrence of IH after the follow-up threshold for hernia detection.

Only one study [8] reported on the operation time (OT) needed for mesh reinforcement, with a mean OT of 198, 213 and 228 min in the PS, onlay and sublay group. Furthermore, a study by Bali et al. comparing PS versus OMR stated a significantly prolonged OT with 131 versus 181 min, respectively. Therefore, regarding the results of this meta-analysis concerning the effect of PMR on IH, a prolongation of surgery is not justified.

This systematic review is limited by the heterogeneous mesh types and techniques used to prevent incisional hernia. After 2010, there was an international effort to summarize and evaluate the existing evidence to determine the optimal closure technique and the use of PMR after median laparotomies [1, 8, 19, 20, 33, 41]. Especially, the suture to wound ratio of 4:1 and the use of a slowly absorbable running suture have a significant influence on the prevention of an IH [41]. Only one of the five included studies in this meta-analysis was performed after these findings and, therefore, used the described accepted gold-standard for abdominal wall closure [8]. This leads to an inevitable limitation of this meta-analysis.

The results of this meta-analysis regarding hernia formation and the effect of PMR stand in direct contrast to the recommended use in patients with an elevated BMI. Although there seems to be striking evidence to use PMR, data regarding morbidly obese patients (obesity grade III) is scarce and showed no significant results when comparing PMR with PS. According to the presented analysis, PMR in patients with a BMI of ≥ 27 undergoing general surgery is safe but does not lower the rate of IH.

## Conclusions

The results of the presented systematic review and meta-analysis suggest an obesity-related reversal of the proven positive effect of PMR in regular weight patients, proposing even more adverse effect of obesity than previously assumed. Seroma formation, the occurrence of SSI and the length of stay showed no significant difference between PMR and PS.
